# Mesenchymal Stem Cell Therapy in Diabetes Mellitus: Progress and Challenges

**DOI:** 10.1155/2013/194858

**Published:** 2013-05-15

**Authors:** Nagwa El-Badri, Mohamed A. Ghoneim

**Affiliations:** ^1^Zewail University of Science and Technology, 6th of October City, Giza 12588, Egypt; ^2^Urology and Nephrology Center, Mansoura 35516, Egypt

## Abstract

Advanced type 2 diabetes mellitus is associated with significant morbidity and mortality due to cardiovascular, nervous, and renal complications. Attempts to cure diabetes mellitus using islet transplantation have been successful in providing a source for insulin secreting cells. However, limited donors, graft rejection, the need for continued immune suppression, and exhaustion of the donor cell pool prompted the search for a more sustained source of insulin secreting cells. Stem cell therapy is a promising alternative for islet transplantation in type 2 diabetic patients who fail to control hyperglycemia even with insulin injection. Autologous stem cell transplantation may provide the best outcome for those patients, since autologous cells are readily available and do not entail prolonged hospital stays or sustained immunotoxic therapy. Among autologous adult stem cells, mesenchymal stem cells (MSCs) therapy has been applied with varying degrees of success in both animal models and in clinical trials. This review will focus on the advantages of MSCs over other types of stem cells and the possible mechanisms by which MSCs transplant restores normoglycemia in type 2 diabetic patients. Sources of MSCs including autologous cells from diabetic patients and the use of various differentiation protocols in relation to best transplant outcome will be discussed.

## 1. Introduction

Obesity and diabetes are the major health challenges for the twenty first century, according to a recent report by the International Diabetes Foundation. The connection between obesity and diabetes is evident in sharing the same risk factors and the fact that 80–90% of type 2 diabetics are also obese. Diabetes is one of the top ten leading causes of death worldwide, according to a recent WHO report [1]. The global prevalence of diabetes in 2012 was estimated to be more than 10% among adults. Of the diabetic population 95% are of type 2, and onset is mainly at an adult age (more than 25 years), with the highest prevalence in the Eastern Mediterranean region and the Americas. Diabetes is the leading cause of renal failure and blindness in advanced countries, and the risk of limb amputation is 10 times higher in diabetic patients. In addition, most diabetic patients develop hypertension and cardiovascular diseases, which account for high rates of morbidity and mortality among adult patients. The disease can be initially treated by oral medication, but eventually, some 27% become insulin dependent. Of these, less than one half achieve the recommended HB A1c level [2], since exogenous insulin cannot provide the tight glycemic control exerted by the pancreas-derived insulin secretion. Replacement of and/or improvements in endogenous *β* cell reserves of the *β* cells would be an ideal therapeutic option. 

A milestone in cell-based therapies for diabetes mellitus has been achieved with the application of islet transplantation from cadaveric donors, and the success of the Edmonton protocol back in 1999 [3]. Type 1 diabetic patients had their diseased islets replaced by high quality functioning islets of Langerhans from fresh cadaveric donors. Increased insulin production, normal blood glucose levels and normal glycosylated hemoglobin levels, and insulin independence were achieved. A modified glucocorticoid-free immunosuppressive regimen using low dose of tacrolimus and normal dose of sirolimus maintained graft acceptance and prevented rejection. While in the initial series only 10% of patients maintained an insulin independent state by five years [4], current data from several centers suggest that with potent T-depletion induction and maintenance immunosuppression that rates exceeding 50% insulin independence can now be routinely achieved [5]. However this often requires the use of more than one donor to achieve an adequate islet engraftment mass. 

As stem cell therapy gained momentum over the past year, efforts to engineer islet-like cells or insulin producing cells from different types of stem cells have offered an appealing alternative to islet transplants. In principle, stem cell therapy avoids some of the serious drawbacks of islet transplantation, most obviously, the shortage of organ donors. Patients can donate their own stem cells to be expanded and differentiated *in vitro* into islet producing cells. The autologous cells can then be injected back into the patient, thus avoiding possibilities and complications of graft rejection, and/or a requirement for and immune suppressive regimen. While islet transplantation in an allogeneic environment may suffer cell exhaustion, stem cell therapies potentially provide sustained source of insulin uncompromised by the many debilitating side effects of the immune suppressive drugs when the transplant is autologous. Sources for stem cell therapies in diabetes mellitus are multiple, including embryonic stem cells (ESCs), cord blood stem cells, iPSCs (induced pluripotent stem cells), and adult stem cells derived from adult tissues. 

Embryonic stem cells have the highest differentiation potential into insulin secreting cells [6]. Transplantation of ESC-derived *β* insulin secreting cells into diabetic individuals presumably requires less immune suppression since ESCs are not as immunologically potent as allogeneic adult cells. Improved techniques of cell culture and use of combinations of growth factors improved the efficiency of differentiation of ESCs into insulin producing cells to more than 25% [7, 8]. The major concerns with ESC-derived *β* pancreatic cells remain to be the potential to form benign cystic teratoma of the remaining high percentage of undifferentiated cells and the ethical concerns of possible destruction of embryos in the process of collecting the ESCs. Safety studies are still ongoing for ESCs as well as for insulin secreting cells derived from adult induced pluripotent stem cells (iPSCs), both of which have achieved the highest differentiation efficacy thus far into insulin producing cells [9, 10]. 

Hematopoietic stem cells, collected from the bone marrow or peripheral blood, have shown some promise in treating type 1 DM. When transplanted into NOD (nonobese diabetic) mice, HSCs conferred protection from hyperglycemia, and attenuated islet lesions were observed [11]. The role of HSCs in the treatment of type 1 diabetes has been proposed to be achieved via an immune suppressive mechanism. The use of immunosuppressive drugs as a conditioning regimen prior to HSC transplantation may have contributed to alleviate the autoimmune reaction and achieve improvement in the levels of insulin, hyperglycemia, and the levels of C-peptide in diabetic patients [12]. A small scale prospective study of type 2 diabetic patients undergoing autologous bone marrow stem cell transplantation on the treatment of insulin-dependent type 2 DM with severe progressive insulin dysfunction showed promising results on the safety of the stem cell protocol and a noticeable reduction of insulin requirements. In this study, Bhansali et al. used autologous bone marrow-derived SCs without separation into HSCs or MSCs. The cells were directly injected into the pancreas via the gastro-duodenal artery. It was not clear if these cells helped in regeneration of the *β* islet cells or they formed insulin producing cells in situ [13]. However, large scale studies with more diverse patient populations and long-term followup are still required to determine the feasibility of such an approach. 

A different study used highly proliferative progenitor cells obtained from fetal liver to treat insulin dependent diabetes. Fetal liver progenitor cells were genetically manipulated to express the pancreatic duodenal homeobox 1 (Pdx1) gene, a key regulator in insulin secretion [14]. Differentiated fetal liver progenitors were transplanted into NOD diabetic mice, the animals became euglycemic, and their serum showed increased levels of human C-peptide.

Some of the successful stem cell transplant protocols maintained that activation of endogenous progenitor cells, rather than differentiation of donor cells into insulin producing cells, provides reasonable mechanistic justification for the improved insulin secretion. The search for endogenous pancreatic stem cells has not been promising, but a recent study by Banakh et al. identified and isolated stem cells from the adult pancreas expressing Pdx1 transcription factor required for pancreas development and *β* cell function. Culture of pancreatic stem cells in serum-free media and in the presence of defined growth factors resulted in differentiation into insulin producing cells. Insulin production was maintained after transplantation of differentiated cells grown in vascularized chambers into diabetic mice. Pancreatic injury seems to have stimulated the stem cells' ability to secrete insulin and increases their number [15]. 

## 2. Experiments with MSCs

MSCs derived from a variety of human adult tissues were utilized in an attempt for their differentiation into insulin producing cells. Bone marrow [16–18], adipose tissue [19], umbilical cord or its blood [20, 21], fibroblasts [22], endometrium [23], and liver cells [24] are among several others. The richest source for MSCs, however, is the bone marrow. Bone marrow MSCs have several advantages when used for the purpose of tissue repair. They have a high capacity to self-replicate and differentiate, both* in vitro* and* in vivo*, to bone and fat-forming cells and other tissue cells. They maintain the capacity of multilineage differentiation potential, both within and across lineage barriers. They are easy to cultivate and expand, and maintain pluripotentiality after prolonged culture conditions [25]. MSCs are also preferred for transplantation purposes since they are considered immunologically “inert” and possess immune suppressive qualities [26] which make them ideal candidates when a fully matched donor is not available. When transplanted along with other adult stem cells, mesenchymal stem cells facilitated engraftment of hematopoietic stem cells across lineage barriers both in mouse models and clinical trials [27, 28]. Although the high differentiation potential of ESCs and iPSCs clearly surpasses that of adult stem cells, including MSCs, the latter remained a favorite for transplantation scientists because of their “benign”, nontumorigenic differentiation potential. In effect, MSCs have been used to inhibit tumor growth *in vivo* [29].

Many protocols were tried to differentiate MSCs into insulin producing cells [30]. Cultures in media with high glucose content were a common feature. However, some authors [31] argued that the presence of insulin in such cells does not indicate intrinsic insulin production and suggested that insulin from the utilized culture media can be absorbed by and sequestrated in these cells. In order to establish persuasive proof of concept, several criteria have to be met [32]. These include coexpression of insulin and C-peptide by the same cells with demonstration of insulin storage granules. Specific gene expression similar to those of pancreatic *β* cells have to be identified. There should be a stepwise increase in insulin and C-peptide release as a function of increasing glucose concentrations *in vitro*, cure of hyperglycaemia following cell transplantation in diabetic animals, and prompt return of diabetes when these cells are removed. Evidence should also be provided that no regeneration of islets in streptozotocin diabetic animals had occurred [32].

Determination of a consistent protocol for differentiating stem cells into insulin producing cells has been more successful with ESCs than adult cells, including MSCs. Choi et al. have reported that bone marrow MSCs could be induced to differentiate *in vitro* into insulin producing cells using undefined growth medium [33]. In these experiments, injury by pancreatectomy was used to simulate the production of “regenerative” rat pancreatic extract. Insulin-producing islet-like clusters were generated after coculture of MSCs in the presence of rat pancreatic extract. Functional insulin producing cells were responsive to normal glucose challenge and were deemed useful for potential diabetes therapy. Similarly, insulin positive cells were produced from rat bone marrow stromal cell [34]. Oh et al. used more defined differentiation conditions to induce insulin producing cells from adult marrow stromal cells [35]. These islet-like aggregates demonstrated both gene expression patterns similar to those of *β* cells, active production of insulin, and lowered circulating glucose levels when transplanted under the kidney capsule. Nephrectomized mice quickly relapsed and died within a few days of the removal of the islet graft.

The use of autologous stem cells for the treatment of type 2 diabetes is central to the cell therapy for diabetic patients. The position of the medical community on the use of HSCs, whether autologous or allogeneic for the treatment of DM, is considered investigative and not medically necessary, mainly because of the use of either ablative or nonmyeloablative immune suppressive regimens. Progressively type 2 diabetic patients usually suffer complications and metabolic and immunologic consequences of the disease that render them poor candidates or aggressive stem cell therapy. The use of autologous MSCs represents an attractive, gentler alternative to allogeneic stem cell transplants and autologous HSC transplant. To determine whether autologous MSCs from diabetic patients could differentiate into insulin producing cells, Sun et al. successfully developed a protocol to differentiate diabetic MSCs into insulin producing cells [36]. However, it was not clear whether these cells were superior to normal MSCs collected from control, nondiabetic individuals, neither did the study address the question to the molecular mechanism for the marked ability of stem cells collected from type 2 diabetic patients to produce insulin. 

In addition to the use of growth factors, genetic manipulations of MSCs may represent an effective strategy to generate large numbers of insulin producing cells. MSCs that express Oct 4 embryonic marker and have long telomeres were transfected with murine IPF1, HLXB9, and FOXA2 transcription factors involved early in the endocrine developmental pathway. Genetic manipulations of naive marrow MSCs using viral vectors promoted expression of insulin and key transcription factors of the endocrine pancreas development pathway [37]. The development of the iPSC cells without viral vectors helped the generation of pluripotent cells from an autologous source [38]. In this case, immunosuppression is not needed. However, the possibility of a tendency for tumorigenicity still exists.

Other factors that seem to affect the differentiation potential of MSCs into insulin producing cells include the source of MSCs, which seems to play an important role in their response to differentiation factors. Bone marrow derived MSCs, for example, have shown to be more potent than adipose tissue derived MSCS to differentiate into insulin producing cells [39]. In this regard, stem cell transplant may result in sustained euglycemia disproportionate to the number of injected cells. Phadnis et al. showed that further* in vivo* differentiation into insulin producing cells may account for this effect [40]. 

## 3. How MSC Transplantation Contributes to Treatment of Diabetes

Earlier attempts to treat diabetes using stem cells assumed differentiation of ESCs into insulin secreting *β* cells, rather than other microenvironmental modifications, to treat diabetic symptoms and restore normoglycemia [41–43]. However, factors that could potentially contribute to normoglycemia, such as immune suppression and anti-inflammatory effects, were not regularly considered or rigorously assessed. When evaluating the mechanism of action of MSCs in treatment of diabetes, many differences between MSCs and ESCs, the primary prototype for stem cell therapy, should be considered. First, MSCs are less pluripotent, already more differentiated than ESCs, rendering the efficacy of differentiation into insulin secreting cells more challenging. Transdifferentiation of MSCs into insulin secreting phenotype has been shown by some laboratories to follow a dedifferentiation followed by redifferentiation pathway [44]. Other laboratories challenged this model, given that MSCs are of mesodermal origin while *β* cells are of endodermal origin [33, 45]. Second, unlike ESCs, MSCs can be obtained from different sources, and frequently of nonhomogenous lineages, like bone marrow, adipose tissue, pancreatic stroma, and human umbilical cord blood. Growth factor cocktails designed to induce effective differentiation of ESCs into insulin producing cells have been well-characterized [8]. This is not the case with MSCs. While bone marrow MSCs, for example, can respond to nicotinamide, betacellulin, and other growth factors stimulation and incur about 5% transdifferentiation success into insulin-producing cells, adipose derived MSCs fail to reach such modest efficiency [19, 46]. Umbilical cord MSCs on the other hand have different growth and differentiation patterns than bone marrow MSCs. While the latter naturally differentiates into fat and bone cells *in vivo* and in culture, the former rarely produces adipose tissue under the same culture conditions [46]. Because of the fetal origin of cord blood, placenta, amniotic fluid, and Wharton's jelly-derived MSCs, they are much similar to ESCs than marrow MSCs, as they express embryonic markers and endoderm lineage markers [22, 47]. Theoretically, this resemblance to embryonic cells should render fetal cells more superior in differentiation into insulin producing cells; however, this has not been the case. Efforts to coax cord blood and other fetal MSCs to differentiate into insulin producing cells in several laboratories have not been satisfactory [17, 48, 49].

The third difference between MSCs and ESCs is that MSCs can be obtained from the patient for autologous transplant. This of course can also be the case for ESCs if reproductive cloning techniques are followed; however, autologous MSCs from diabetic patients are still remarkably different from ESCs, because of prolonged exposure to hyperglycemia. Studies in transgenic mice showed that stem cells engineered to produce insulin did much more efficiently in hyperglycemic environment [18, 50, 51]. Studies from Ghoneim's laboratory [16], which were the first to compare MSCs from diabetic and nondiabetic patients, found no compromised insulin secreting ability in MSCs from diseased patients. There were no significant differences in the characteristics of growth, differentiation, or gene profiles of the MSCs collected from diabetic or nondiabetic donors. To the contrary, the insulin secretion profile of MSCs obtained from nondiabetic donors was significantly less than that of diabetic donors. Indeed, high glucose concentration in the MSC culture was considered as a potent inducer for pancreatic islet differentiation [52]. Unique factors present in the diabetic microenvironment may account for these differences [40]. On the other hand, impaired therapeutic capacity of autologous stem cells in a model of type 2 diabetes had been reported [53]. Nevertheless, assays are now available to predict the potency of MSCs for their therapeutic selection [54].

While unorthodox in the view of several studies that reported defective microenvironment in autoimmune disease [35, 55], type 2 diabetes mellitus is a metabolic disorder, and insulin malfunction is only achieved in advanced disease. Metabolic effects of hyperglycemia on MSCs, rather than inherent genetic malfunction, seem to determine this preferential ability of patient derived MSCs to respond favorably to hyperglycemia. 

Forth, MSCs are niche cells. Their traditional role in the bone marrow is the formation of the stroma and facilitation of growth, differentiation, and engraftment of HSCs. It is worth examination whether this niche role is contributing to the normoglycemic effect following their transplant. Do they provide the supportive stroma for endogenous islet cells, or stimulate dormant host stem cells to differentiate into insulin producing cells? And how do their immune suppressive qualities contribute to this role? Consensual reports on function of human MSCs transplanted in mice or rats indicated that human, and not murine insulin was elevated in the transplanted mice [16], confirming that the source of insulin is exclusively human donor cells. However, in many of these experiments discrepancy between low insulin levels and achievement of normoglycemia may suggest that factors other than de novo human insulin secretion contributed to the restoration of normal glucose levels. A recent study showed that improved insulin sensitivity, and not secretion accounted for normoglycemia in mice transplanted with MSCs. There were increased cellular AKT phosphorylation and improved signaling in the liver, muscle, and adipose tissue of the recipients of the autologous MSC transplant [56]. Fifth, MSCs, although capable of inducing graft rejection when transplanted cross allogeneic MHC barriers, are considerably more immunologically inert than ESCs. This is especially important in type 2 DM, where a proinflammatory milieu exacerbates the disease*. In vitro *experiments showed that coculture of MSCs with pancreatic islets prevented *β* cell apoptosis induced by treatment with proinflammatory cytokines, TNF *α*, and interferon *γ* [57]. MSCs promoted islet survival and engraftment and reduced inflammation. 

Sixth, unlike highly tumorigenic undifferentiated ESCs, the MSC administration for the treatment of diabetes seems to be effective even without a predifferentiation protocol. Transplantation of undifferentiated MSCs into streptozotocin-induced diabetes in C57Bl/6 mice induced normoglycemia and reversed glycosuria. This was accompanied by improved renal function and histological evidence of regeneration of normal beta pancreatic islets [58].

## 4. Conclusions and Future Perspectives

Replacement of diseased pancreatic islets by healthy functioning ones from cadaveric donors is a highly effective approach to treat insulin dependent diabetes. Careful usage of combined therapies of immune suppressive regimens prolongs graft survival and improves insulin levels and norm glycemic outcome. However, this therapy is hampered by shortage of donors, the need for fresh graft, usually within 8 hours after death, the need for tissues from more than one donor for one recipient, and the incidence of graft rejection. Stem cell therapy, especially using adult MSCs as a source for engineered insulin secreting cells, is considered the next frontier for diabetes treatment. The key to an impressive outcome with stem therapy is to use high quality, well differentiated cells, with evidence of insulin production *in vivo* and sustained normoglycemia. The clinical feasibility of using autologous MSCs along with minimum or no immune suppressive regimens improves the outcome substantially, since most fatal complications in stem transplant recipients are caused by the use of immunosuppression. 

Therapeutic applications in the clinical setting using MSCs depend on their safety and efficacy, both of which have not been yet optimized for type 2 diabetic patients. Safety issues are related to the potential pathogenicity of the cell inoculum. MSCs are currently cultured using fetal bovine serum, which can induce xenogeneic and allergic reactions in transplanted patients, in addition to transmission of xenogeneic pathogens that may contaminate the serum. The immune characteristics of MSCs have been generally encouraging for transplantation purposes; however, there are some reports on the increased tumor formation in animals due to the immune suppressive effects of MSC transplants, particularly in the allogeneic setting [59, 60]. Furthermore, frequent *in vitro* passaging and the long time required for effective differentiation into insulin producing cells can induce mutations and transformations and render the graft unsafe for clinical usage [61]. Safety studies must go hand in hand with efficacy studies to ensure safe long term effects of the MSC transplant. 

Increasing the number of insulin producing cells and their sustained survival is a high priority in stem cell research. If surrogate *β* cells could be obtained in sufficient numbers, two additional questions have to be addressedas follows. For how long can these cells maintain their active function *in vivo*? And what is the optimal site for their transplantation? Information regarding the duration of active function is limited by the observation period following transplantation in experimental models, the longest of which is in order of three months before the animals are sacrificed [16]. It is abundantly clear that experiments with larger animal models and for more extended periods are required.

It is reasonable to assume that experiences with possible sites for pancreatic islet transplantation can be also applied to insulin producing cells derived from MSCs. The liver is currently the site of choice for clinical islet transplantation. However, it is now recognized that it may not provide the optimal microenvironment due to immunologic, anatomic, and physiologic factors that contribute to early loss of the islet mass after its infusion [44]. Alternative sites are currently under investigation and are well summarized in some recent reviews [45, 62, 63]. An interesting approach is transplantation into striated muscles [64]. Striated muscles are easily accessible and have been used for autotransplantation of the parathyroid glands. Moreover, it is almost the only tissue in the adult where physiologic angiogenesis occurs [65]. In their animal experiment, Svensson and colleagues provided evidence that islets grafted into muscle have 3 times more blood vessels than islets at the renal subcapsular site at 2 months aftertransplant [66]. They concluded that the intramuscular site can provide an excellent condition for engraftment. Additional tools may also be needed to improve early graft survival: bioengineered matrices, oxygen carriers, and growth factors.

The potential role of MSCs in the management of several diabetic complications has also been explored. These include their benefit in promotion of healing of diabetic foot [67], control of the onset and/or progression of diabetic nephropathy [68, 69], and attenuation of symptoms of painful diabetic neuropathy [70, 71].
